# Tendon extracellular matrix damage, degradation and inflammation in response to in vitro overload exercise

**DOI:** 10.1002/jor.22879

**Published:** 2015-04-29

**Authors:** Ewa M. Spiesz, Chavaunne T. Thorpe, Saira Chaudhry, Graham P. Riley, Helen L. Birch, Peter D. Clegg, Hazel R.C. Screen

**Affiliations:** ^1^ School of Engineering and Materials ScienceQueen Mary University of LondonLondonUnited Kingdom; ^2^ School of Biological SciencesUniversity of East AngliaNorwichUnited Kingdom; ^3^ Institute of Orthopaedics and Musculoskeletal ScienceUniversity College LondonLondonUnited Kingdom; ^4^ Department of Musculoskeletal BiologyUniversity of LiverpoolLiverpoolUnited Kingdom

**Keywords:** tendon, fatigue, inflammation, matrix degradation, ECM damage, interfascicular matrix, collagen

## Abstract

The role of inflammation in tendon injury is uncertain and a topic of current interest. In vitro studies of tendon accelerated overload damage can serve as a valuable source of information on the early stages of tendinopathy. Viable fascicle bundles from bovine flexor tendons were subjected to cyclic uniaxial loading from 1–10% strain. Immuno‐staining for inflammatory markers and matrix degradation markers was performed on the samples after mechanical testing. Loaded samples exhibited visible extracellular matrix damage, with disrupted collagen fibers and fiber kinks, and notable damage to the interfascicular matrix. Inflammatory markers COX‐2 and IL‐6 were only expressed in the cyclically loaded samples. Collagen degradation markers MMP‐1 and C1,2C were colocalized in many areas, with staining occurring in the interfascicular matrix or the fascicular tenocytes. These markers were present in control samples, but staining became increasingly intense with loading. Little MMP‐3 or MMP‐13 was evident in control sections. In loaded samples, some sections showed intense staining of these markers, again localized to interfascicular regions. This study suggests that inflammatory markers may be expressed rapidly after tendon overload exercise. Interestingly, both inflammation and damage‐induced matrix remodeling seem to be concentrated in, or in the vicinity of, the highly cellular interfascicular matrix. © 2015 The Authors. *Journal of Orthopaedic Research* Published by Wiley Periodicals, Inc. J Orthop Res 33:889–897, 2015.

Tendinopathy is a general term for chronic tendon disease^1^ characterized by a combination of pain, swelling, and impaired tendon performance.^2^ The aetiology of this disease remains to be elucidated and is likely to be multi‐factorial. Tendinopathy is often the result of damage accumulation during overuse, and may also involve either mechanical over‐stimulation or under‐stimulation of tenocytes and the related (imbalanced) remodeling in tendon tissue.^1^ Tendinopathy has often been considered a degenerative disease^1^ not associated with inflammation. However the role of inflammation within the initiation, progression, and resolution of tendinopathy remains unclear.^3^

Tendons have an inhomogeneous multilevel hierarchical composite structure. Considering the mesoscale, looking at the largest hierarchical subunits in tendon, it is possible to describe tendon as a composite material with two main phases (or matrix components). The phases are the fascicles (fascicular matrix, FM), which are longitudinal structures composed predominantly of uniaxially arranged type I collagen fibers, and the interfascicular matrix (IFM) or endotenon that fills the spaces between fascicles (see Fig. 1). IFM is a highly cellular matrix phase consisting of a range of non‐collagenous proteins including elastin, proteoglycans, and also minor collagens.^4^

**Figure 1:**
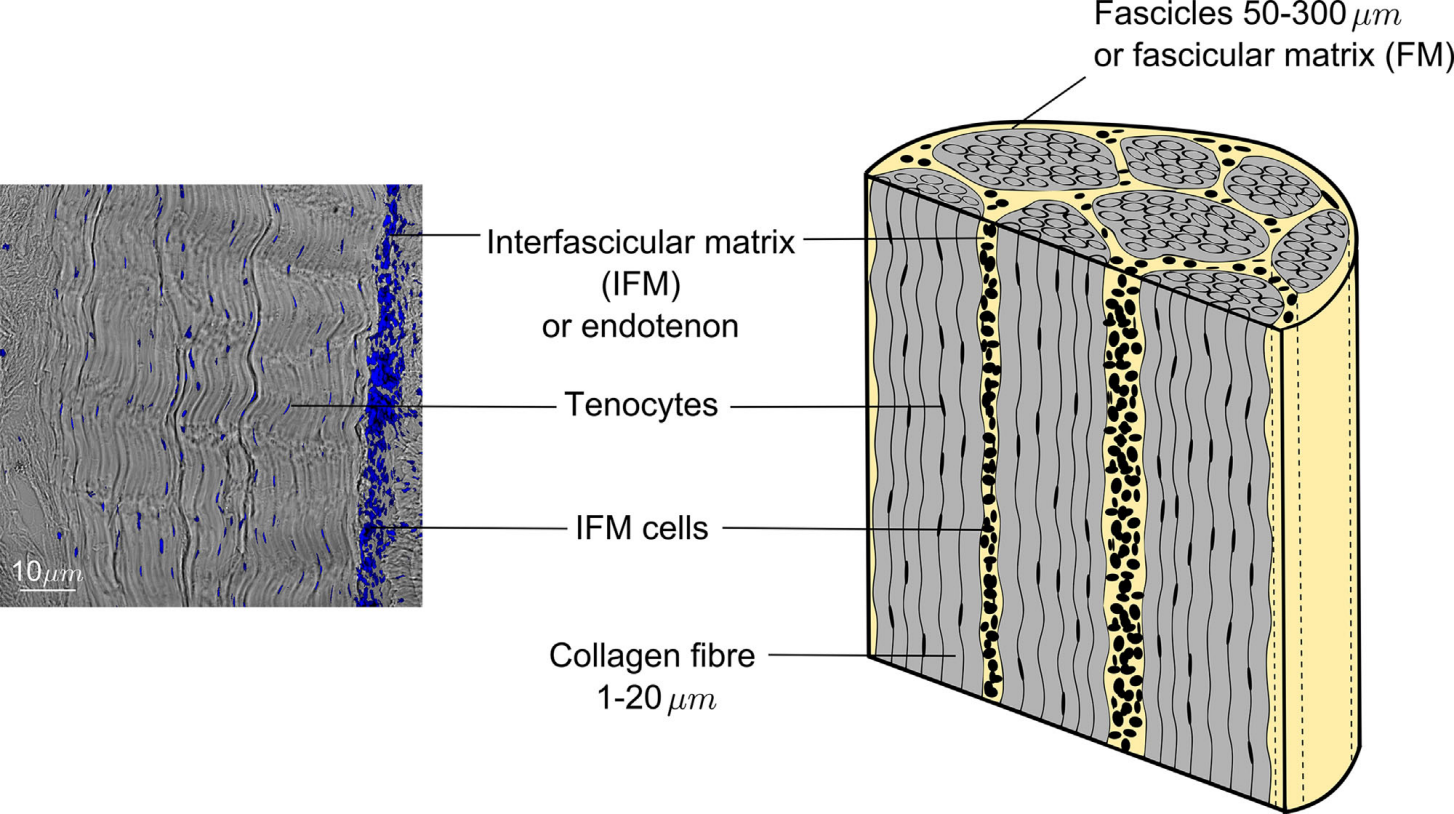
Schematic and histological section of a bovine flexor tendon, depicting the two phases (components) of the tendons structure at the meso‐scale: collagen‐rich fascicles, termed fascicular matrix (FM) and the surrounding endotendon, termed interfascicular matrix (IFM). Notice the different cell shapes and numbers within fascicles (tenocytes) and the IFM (IFM cells). Cell nuclei are stained with DAPI (blue).

The IFM has a more sparse fiber arrangement, with lower volume fractions of fibrillar material than fascicular matrix. Arrangement of fibers in the IFM is more network‐like, which suggests softer, and likely more complex mechanical behavior^5^, ^6^ as compared to the uniaxially arranged, mainly collagen type I, fibers in fascicles. The collagenous phase volume fraction and the orientation of fibers both have previously been shown to have a direct impact on tissue mechanics,^7^, ^8^, ^9^, ^10^ suggesting that the interfascicular matrix is likely more prone to damage and injury. This softer phase is thought to create sliding planes between fascicles that facilitate increased tendon extension and recoil.^11^ However, the exact role of the interfascicular matrix in tendon injury or remodeling remains uncertain.

Tendons are thought to undergo slow remodeling mediated by matrix metalloproteinases (MMPs) and disintegrins and metalloproteinases with thrombospondin motifs (ADAMTS), whose activity is inhibited though tissue inhibitors of metalloproteinases (TIMPs).^1^

These enzymes, and their inhibitors, have roles in extracellular matrix turnover in health but also in pathological conditions. However, it remains unclear how tendon turnover is mediated by the tendon cells population. The cell population in tendon is heterogeneous, consisting of elongated tenocytes between collagen fibers within the FM and IFM cells which are more rounded in shape (Fig. 1). However, there are as yet no specific markers to distinguish these types of cells from each other or from other connective tissue cells.^1^ It has been shown that cell shape is influenced by local mechanical properties,^12^, ^13^ and the two cell populations of tendon likely live in mechanically distinct environments (stiffer FM or softer IFM). In addition, the IFM regions are more highly cellular than FM regions. The turnover of non‐collagenous matrix has been shown to be faster than that of collagenous matrix in tendon. The greater quantity of non‐collagenous matrix in the IFM thus suggests that IFM cells may be more active in healthy turnover, but they may also play different roles in tendon injury and repair.^14^

The role of an inflammatory response in tendinopathy has been discussed in the literature, but no consensus has been achieved. How the inflammation results in damage accumulation and the initiation of tendinopathy is not easily investigated in human subjects, because the affected tendons can normally only be assessed scientifically once tendinopathic changes are advanced. Therefore, in vitro studies of the inflammatory response to induced tendon overload damage can serve as a valuable source of information on the early stages of tendinopathy. The goal of this project was to investigate the influence of cyclic overload exercise performed in vitro on the expression of inflammatory mediators and markers of matrix degradation in bovine tendons comparing the response of FM and IFM regions. We hypothesized that cyclic loading would result in an increase in the levels of the inflammatory markers COX‐2 and IL‐6, and would also increase levels of the matrix degrading enzymes MMP‐1, ‐3, and ‐13, resulting in increased collagen degradation. Further we hypothesized that the response to overload would be localized predominantly in the IFM.

## METHODS

### Samples and Loading Protocol

**Figure 2:**
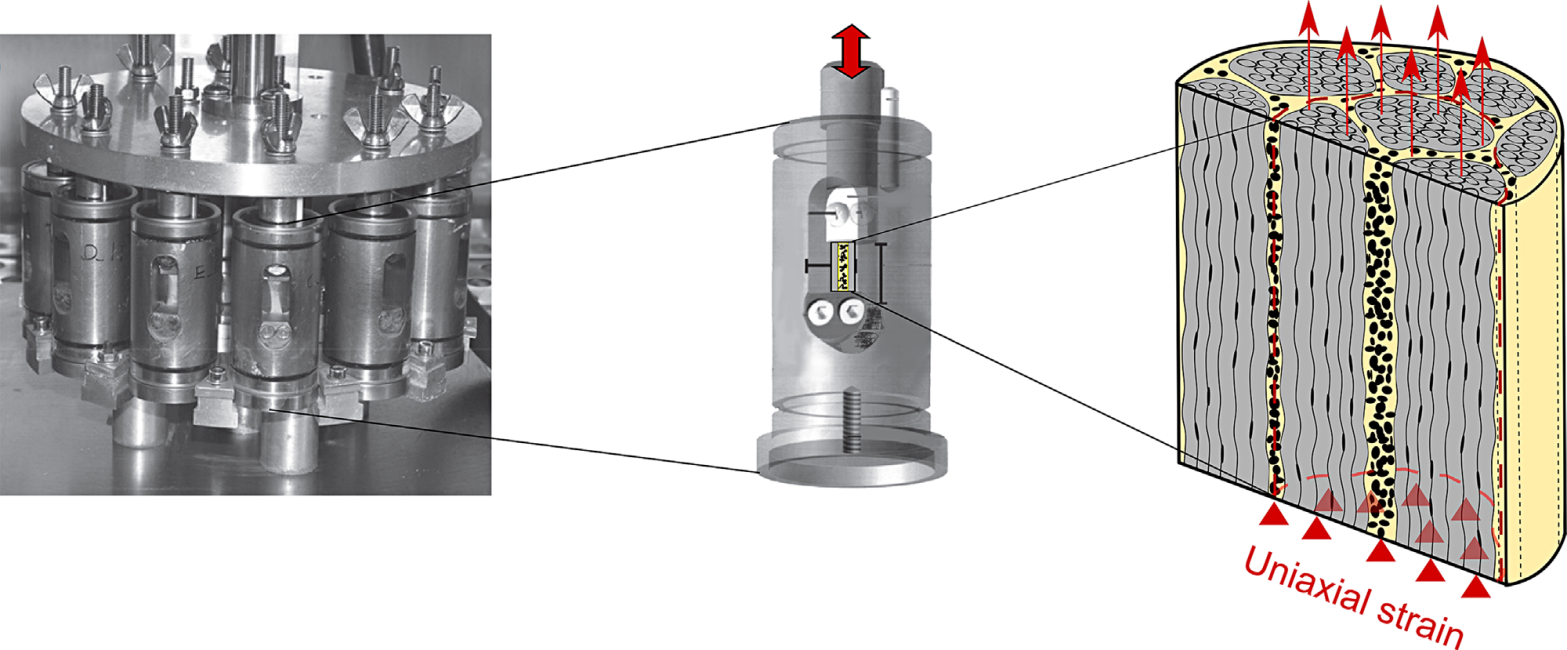
Loading system used to apply overload exercise to fascicle bundles.^16^ Test samples contain collagen‐rich fascicles (gray) and interfascicular matrix (yellow) which allows for later observation of the both regions' response to overload exercise.

Deep digital flexor tendons from the forelimbs of skeletally mature adult cows aged 18 months (*n* = 3) were obtained from an abattoir immediately post‐mortem. As we aimed to investigate the matrix response from both collagen‐rich fascicles and the interfascicular matrix, fascicle bundles (*n* = 12) containing both types of matrix were dissected (Fig. 2). The fascicle bundles we tested in this study were on average 1.5 mm in diameter, incorporating a minimum of three fascicles. From each tendon, one fascicle bundle was assigned to each test group:

1. CON ‐ fresh control: snap frozen immediately after dissection;
2. STA ‐ static control: held at 1% strain for 24h;
3. 300C ‐ cyclically loaded for 300 cycles (5min) from 1–10% uniaxial strain (1Hz) followed by 1% static strain for 24h;
4. 1,800C ‐ cyclically loaded for 1,800 cycles (30min) from 1–10% uniaxial strain (1Hz) followed by 1% static strain for 24h.

Samples from groups 2–4 were placed into individual chambers of the multiple chamber system (Fig. 2), gripped with a gauge length of 10 mm. The cyclically loaded samples (groups 3–4) were subjected to large cyclic strains from 1–10%, using a Bose mechanical test system (Wintest v 4.0, Bose Coorporation, USA) housed in an incubator. The straining regimes have previously been shown to simulate overload.^15^ During testing the samples were maintained in sterile cell culture medium (DMEM) supplemented with penicillin (50 U/ml), streptomycin (0.05 mg/ml) and l‐glutamine (2 mM) and maintained in an incubator at 37 °C, 20% O_2_, 5% CO_2_.

After the end of the 24 h test period, samples were removed from the chambers and snap frozen in optimal cutting temperature (OCT compound, VWR) embedding medium (cooled in hexane on dry ice) and stored at −80 °C. Three 20 µm thick longitudinal cryo‐sections were cut from each sample and mounted on poly‐l‐lysine coated slides for further analysis.

### Immuno‐staining

Dual immuno‐staining for the following three marker pairs was performed:

- inflammatory markers ‐ cyclooxygenase (COX‐2) and interleukin (IL‐6).
- matrix degradation markers: matrix metalloproteinase MMP‐1 and collagen degradation marker C1,2C.
- matrix degradation markers: matrix metalloproteinases MMP‐3 and MMP‐13.

Details of the antibodies used are shown in Table 1.

**Table 1:** Details of the Antibodies Used

Marker	Target	Primary antibody	Concentration	Secondary antibody	Concentration	Validation
IL‐6 Interleukin‐6	Inflammatory marker	Goat polyclonal anti‐IL6 (R&D systems AF1886)	1:25	Donkey anti‐goat 543 (red)	1:500	16
COX‐2 Cyclo‐oxygenase‐2	Inflammatory marker	Mouse monoclonal anti‐Cox‐2 (Cayman Chemical 160112)	1:50	Donkey anti‐mouse 488 (green)	1:500	17, 18
MMP‐1 Matrix metallo‐proteinase‐1	Fibrillar collagens degradation	Goat polyclonal anti‐MMP1 (Santa Cruz sc‐6837)	1:50	Donkey anti‐goat 543 (red)	1:500	19
C1,2C	Collagen type I and II degradation	Rabbit polyclonal anti‐C1,2C (Abcam 18898)	1:20	Donkey anti‐rabbit 488 (green)	1:500	20, 21
MMP‐3 Matrix metallo‐proteinase‐3	Minor matrix proteins degradation (collagens III, IV, IX and X, proteoglycans, elastin)	Goat polyclonal anti‐MMP3 (Abcam 18898)	1:25	Donkey anti‐goat 543 (red)	1:500	22
MMP‐13 Matrix metallo‐proteinase‐13	Fibrillar collagens degradation	Rabbit polyclonal anti‐MMP13 (Santa Cruz sc‐30073)	1:50	Donkey anti‐rabbit 488 (green)	1:500	23

Both the inflammatory markers selected, IL‐6^24^, ^25^ and COX‐2^26^, ^27^ have previously been shown to be upregulated in tendinopathy. MMPs 1 and 13 were selected as they are known to be active against fibrillar collagens, including the predominant collagen type I.^1^, ^28^, ^29^, ^30^ MMP‐3 was selected as it degrades other minor proteins in tendon matrix, including collagens III, IV, IX, and X, and also proteoglycans and elastin.^28^, ^29^, ^30^, ^31^, ^32^, ^33^ The C1,2C (or COL 2 3/4C short) antibody detects the carboxy terminus of fragmented type I and II collagen, cleaved by collagenases MMP‐1, MMP‐8 or MMP‐13.^21^, ^34^, ^35^, ^36^, ^37^

Cryo‐sections were thawed and fixed with acetone and then rehydrated in phosphate buffered saline (PBS, Sigma–Aldrich) followed by tris buffered saline (TBS, Sigma–Aldrich, Dorset, United Kingdom) plus 0.1% Triton X‐100 (Sigma–Aldrich, Dorset, United Kingdom) to permeabilize cell membranes. Blocking buffer‐TBS with 1% bovine serum albumin, (BSA, Sigma–Aldrich, Dorset, United Kingdom) 10% donkey serum (Sigma–Aldrich, Dorset, United Kingdom) was applied to sections for 2 h at room temperature. After draining, the primary antibodies diluted in blocking buffer with 1% BSA were then applied. Concentrations of antibodies used are listed in Table 1. For negative controls, blocking buffer without the primary antibody was applied. Sections were incubated overnight at 4 °C. Next, sections were rinsed in TBS with 0.1% Triton, followed by the application of the fluorophore‐conjugated secondary antibodies, diluted to the concentration listed in Table 1 in blocking buffer, and incubated for 1 h at room temperature. This step was done in the dark to avoid photo‐bleaching. Finally, sections were rinsed in PBS, mounted with DAPI containing mounting medium (ProLong Gold, Life Technologies, Paisley, UK) and left overnight at 4 °C to allow distinction of the highly cellular IFM regions from less cellular fascicular regions. Negative control sections were also imaged, to confirm lack of binding of secondary antibodies.

### Imaging, Image Processing, and Analysis

Sections were imaged with a confocal microscope (Leica TCS SP2) using a 20 × objective and 2 × digital zoom. Within each slide, at least one region was imaged, in an area including fascicular and interfascicular matrix. The region was selected using bright field imaging, ensuring an area incorporating both interfascicular matrix and fascicular matrix were selected, but blinding the selector to the extent of staining for any marker during region selection. Four matching images were taken of each region: bright field; blue channel (wavelength 351 nm) for visualization of DAPI showing cell nuclei; green channel (wavelength 488 nm); and red channel (wavelength 543 nm) used for each pair of dual antibodies investigated.

Semi‐quantitative analyses of the images were performed in Gimp 2.8 software (GNU Image Manipulation Program). Images were thresholded at a single level threshold (for a given stain) and overlaid for visualization and to facilitate a qualitative assessment of the extent and intensity of immuno‐staining within IFM and fascicles (FM). All images were assessed for the number of pixels falling above the threshold. The brightness of each pixel falling above the threshold was first measured after which an average image brightness was calculated. Stain intensity was calculated, defined as the stain brightness multiplied by the stained area.^38^

One hundred and eight stained sections have been imaged and evaluated from which mean stain intensity and standard deviation was determined for each antibody used across all load conditions. Some images were discarded based on features like prominent blood vessels in the field of view. Sections with a bright background or large artefactual dots visible across both green and red channels (possible dirt particles) were also discarded.

## RESULTS

### Tendon Matrix Damage

**Figure 3:**
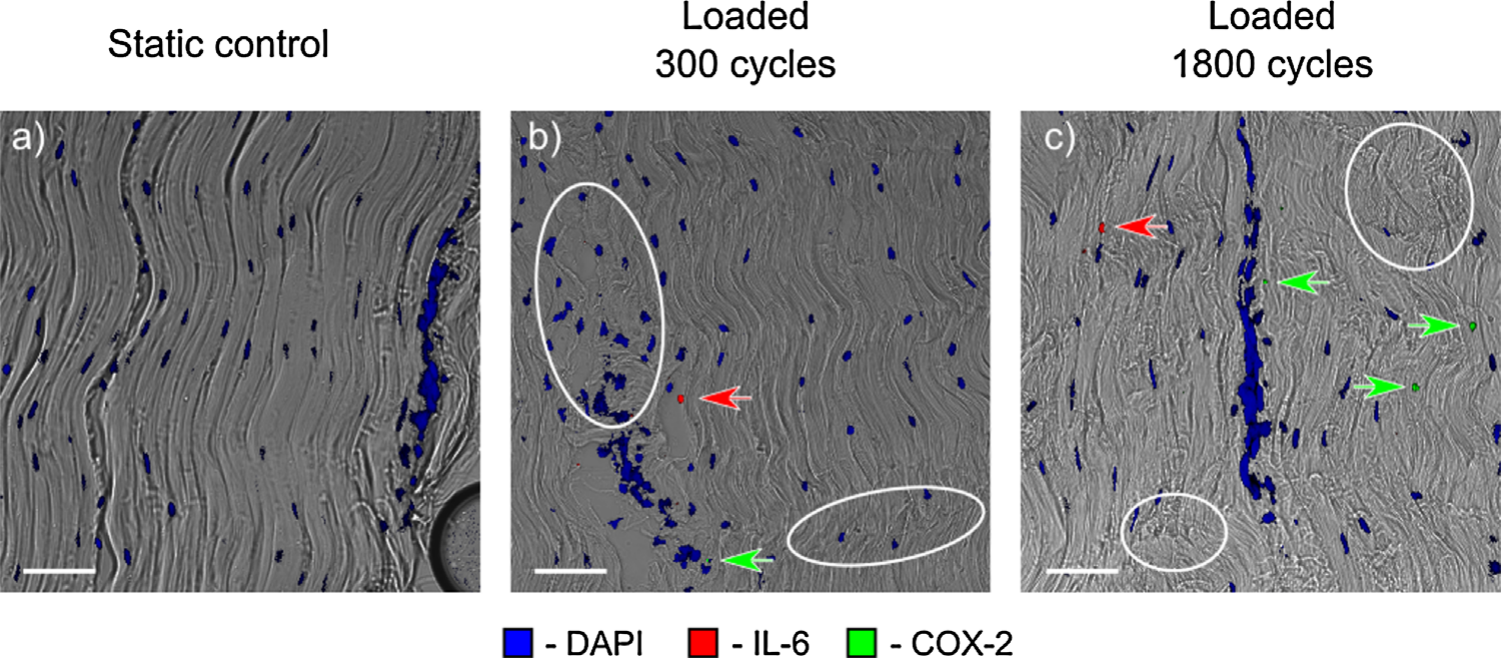
Typical static (a) and loaded (b, c) samples immunostained for inflammatory markers. Damage is evident in both the fascicles and the interfascicular matrix of the loaded samples (examples of damage— collagen fibers kinks and IFM disruption shown with white ellipses). Both inflammatory markers were only expressed in loaded samples, with COX‐2 expression increasing with the cycle number. Scale bar is 10 µm.

**Figure 4:**
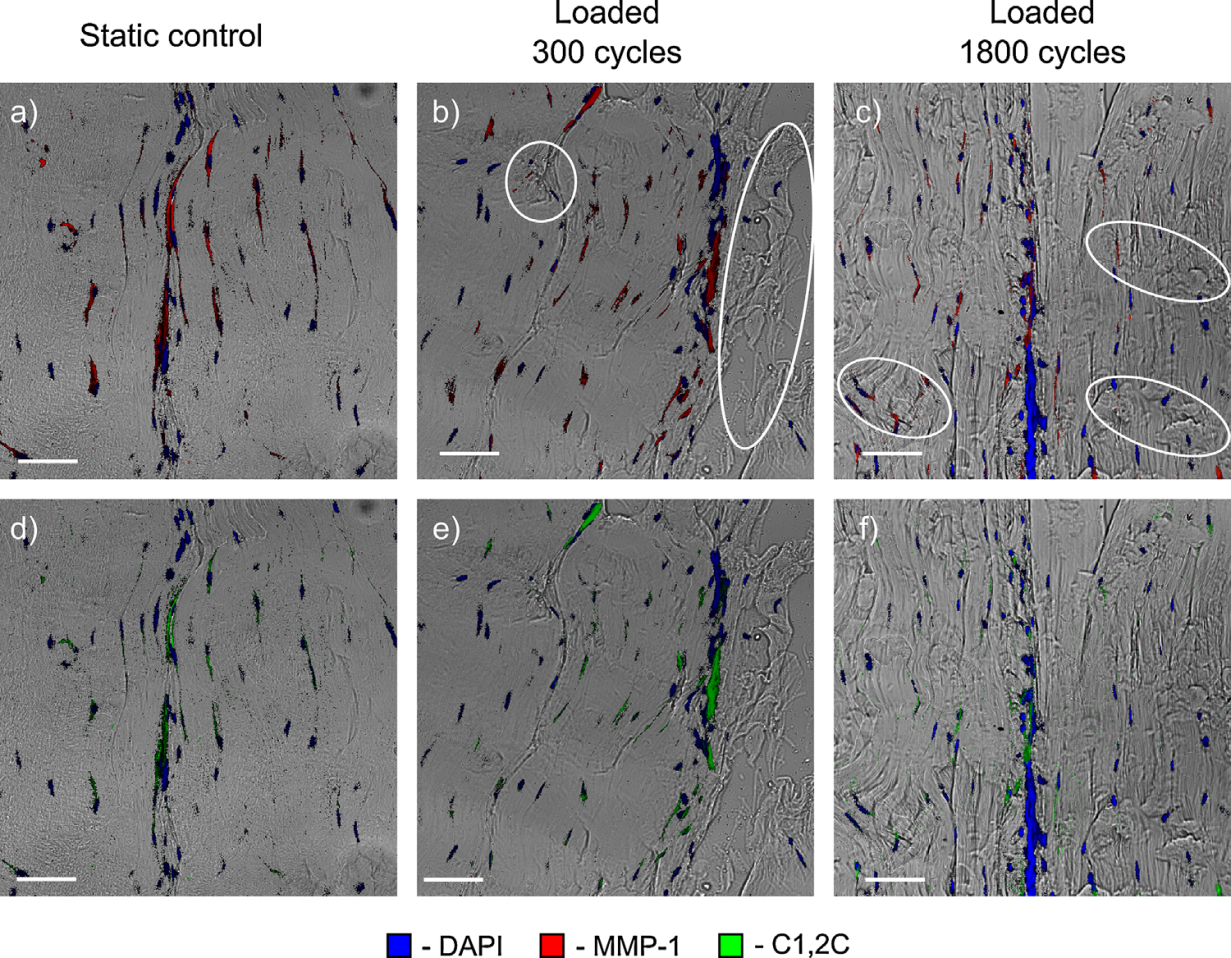
Typical static (a, d) and loaded (b, c, e, f) samples immunostained for collagen matrix degradation markers. MMP‐1 and C1,2C are colocalized and associated predominantly with the interfascicular matrix, in both control and loaded samples, indicating continual turnover of the interfascicular matrix. Damaged areas of fibers, fiber kinks, and the IFM damage are marked with white ellipses. Scale bar is 10 µm.

**Figure 5:**
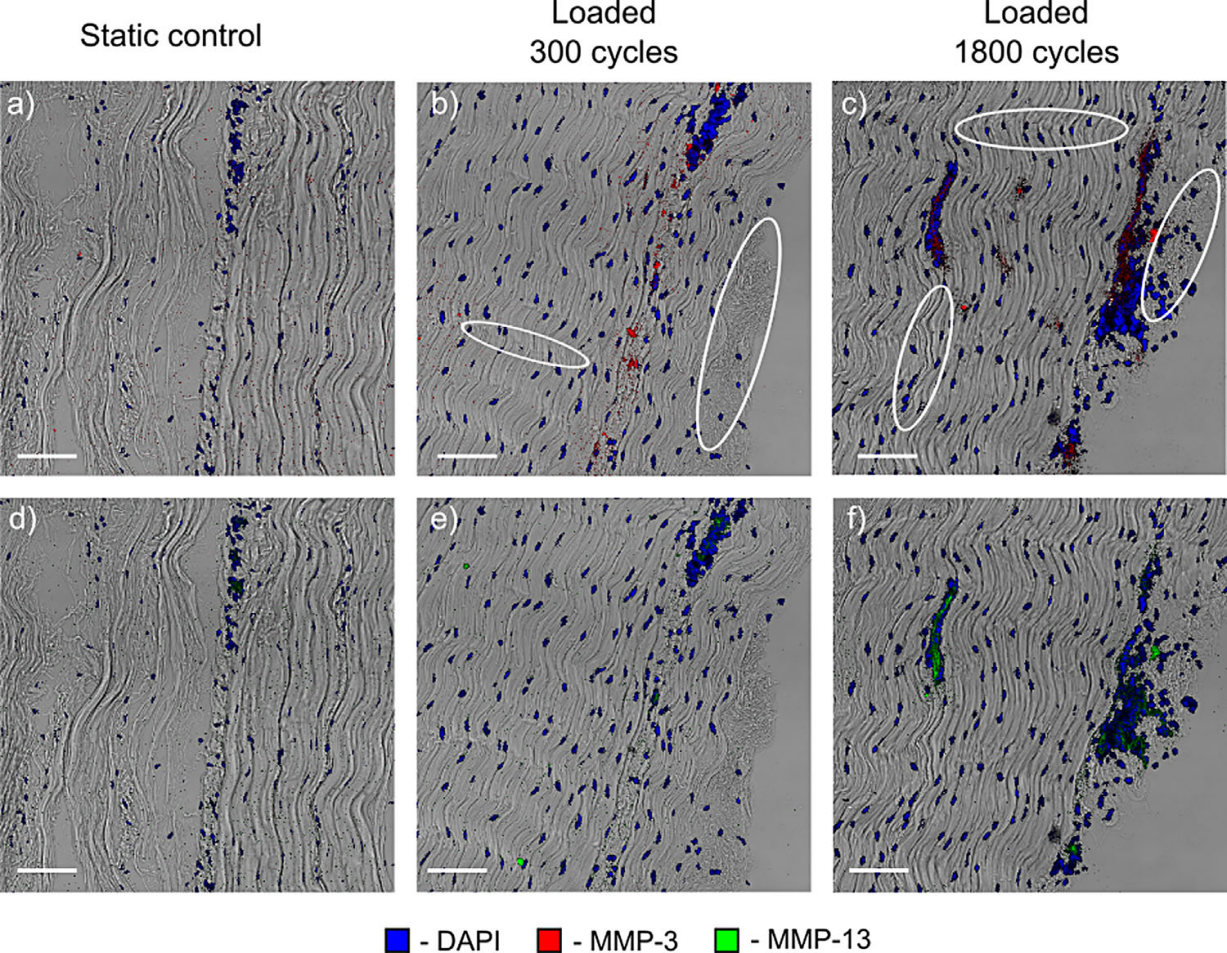
Typical static (a, d) and loaded (b, c, e, f) samples immunostained for matrix degradation markers. MMP‐13 is associated with areas in which there is a large aggregation of tenocytes and in the interfascicular matrix. MMP‐3 expression levels are higher and also remain mainly related to interfascicular matrix. Damaged areas of fibers and the IFM are marked with white ellipses. Scale bar is 10 µm.

Signs of fibrils damage were visible in sections of tendons that were loaded for either 300 or 1,800 cycles (marked with white ellipses in Figures Fig. 3, Fig. 4,3, 4 and 5). fiber kinks and damage were visible and increased in severity with increasing load regime. Damage was also evident in the IFM as disruption of the structure.

**Figure 6:**
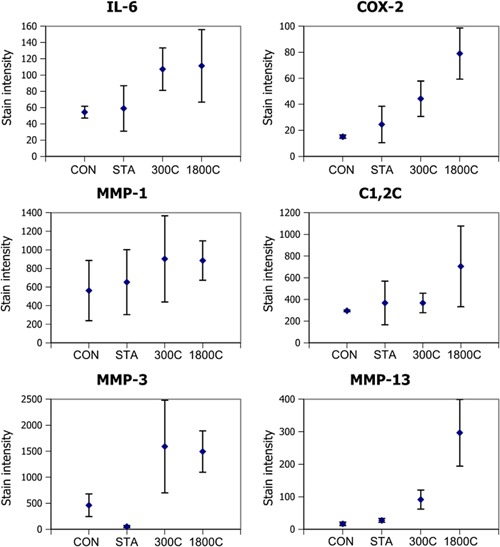
Changes in stain intensity for control samples (CON), statically loaded samples (STA), samples loaded for 300 cycles (300C) and samples loaded for 1,800 cycles (1,800C) calculated as an average across the whole sample. Test groups show the mean values for the stain intensity (defined as stain brightness multiplied by the stained area). Error bars show standard deviations and are shown for all data sets. Note the differences in the levels of stain for different antibodies (*y*‐axes), with the highest levels of expression for MMP‐3 and the lowest for the inflammatory markers (IL‐6 and COX‐2).

A semi‐quantitative evaluation of the inflammatory and matrix degradation markers was carried out, as summarized in Figure 6 and discussed below.

### Inflammatory Markers

Inflammatory markers were only expressed in the cyclically loaded samples (see Fig. 3) with staining seen within both the FM, and IFM.

The intensity of IL‐6 staining was similar between the samples loaded for 300 and 1,800 cycles, while the areas of COX‐2 staining (see Fig. 3) increased substantially with increasing time of loading.

### Matrix Degradation Markers

The matrix degrading enzyme MMP‐1 and degradation marker C1,2C were present in control samples (Fig. 4 a and d) as well as the loaded samples (Fig. 4 b, c, e, f), with the high levels of these markers in controls suggesting continuous tendon turnover.

MMP‐1 and C1,2C were colocalized in many areas therefore they are shown in separate images in Figure 4. Whilst staining for these antibodies was observed within the FM and often associated with tenocytes, it was predominantly localized to the IFM, suggesting that continual turnover is predominantly localized to the IFM.

MMP‐1 stain intensity (Fig. 6) showed a trend towards increasing with loading, as did the area stained with C1,2C. Standard deviations in MMP‐1 and C1,2C staining data were both high with different tendons showing large variability. However data indicated possible dose dependent staining, suggesting increased collagen breakdown with more overload.

By contrast, little MMP‐3 or MMP‐13 was evident in either control group. However, in loaded samples, some sections showed increased staining of these markers, again localized mainly to the IFM (see Fig. 5). While MMP‐3 staining showed a similar intensity in both loaded groups, the response appeared to be more strain dose dependent for MMP‐13 (Fig. 6).

### Fold Change of Markers With Increasing Loading Time

**Figure 7:**
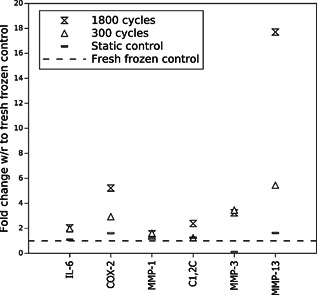
Mean fold change in stain intensity in the loaded samples (static controls, samples loaded for 300 and 1,800 cycles) with respect to fresh frozen controls (dotted line).

Figure 7 presents the mean fold change in stain intensity (stain brightness multiplied with stained area) with respect to the fresh frozen controls (shown by the horizontal dotted line) for each stain and all load conditions. No visible changes were observed between fresh frozen and static control samples for any stain. Fold changes between fresh frozen and static controls were between 1.1 and 1.6 for all stains except MMP‐3, where static controls showed, on average, less staining than the freshly frozen controls.

There are clear fold increases in inflammatory markers with strain in loaded samples: 2.0 for IL‐6 and 5.2 for COX‐2, for the samples loaded for 1,800 cycles (Fig. 7). The highest fold increase of 17.7 was observed for MMP‐13.

Inflammatory markers and matrix degrading enzymes MMP‐3 and MMP‐13 all showed a clear increase with load, suggesting a response to cyclical mechanical loading. By contrast, MMP‐1 and C1,2C were both evident in control samples and showed little change with loading, suggesting the constant turnover is less affected by the range of the overload strains applied in our in vitro experiments.

## DISCUSSION

This study subjects viable bundles of fascicles from bovine flexor tendons to overload damage, adopting immunohistochemistry to investigate the cellular response to injury. The in vitro overload protocol adopted here has previously been validated, to ensure the generation of accelerated matrix damage,^15^ and data indicated a rapid inflammatory response from tendon cells directly after overload. An in vitro model of overuse such as this is unlikely to fully recapitulate the in vivo environment, but allows for precise control of the loading environment, which it is otherwise very difficult to achieve. Further, our in vitro model is likely to result in larger and more uniform straining across our fascicle bundles than would be seen in vivo, where strains are distributed across the whole tendon's hierarchical structure and along the whole length of the tendon. However, this accelerated fatigue model facilitates assessment of the cell response to local tendon damage.^16^

Immuno‐staining and semi‐quantification relies on sufficient antibodies having access to epitope binding sites within the tissue section. The structure of the two phases of tendon: FM and IFM is different, with densely packed collagenous fibers (mainly collagen type I) within the FM and sparser, network‐like fibrillar structure in the IFM. It is feasible that antibody binding epitopes were more accessible in the IFM than in the dense FM structure, which could lead to diminished staining in the FM phase of tendon. Also different antibodies are likely to have different binding affinities, which makes the comparison between different stains difficult. A detailed solution for this problem could be devised with molecular dynamics simulations of the particular biological surface and the immuno‐staining solution, but this was not in the scope of the work presented here.

This study suggests that inflammatory markers may be expressed rapidly and early after tendon overload exercise (within 24 h following 5 or 30 min of overload exercise in our in vitro model), indicating that inflammation may be an early occurrence in tendinopathy. We choose to study IL‐6 as this inflammatory mediator has previously been linked to tendinopathy^24^ and COX‐2 as increased levels of COX‐2 have been shown in patellar tendinopathy,^26^, ^27^ loaded human tendon cells^39^ and injured tendons.^40^ Other markers of inflammation such as IL‐1 were not included in this study but have previously been implicated in tendinopathic tendons^41^, ^42^ or bursa of the shoulder.^25^ In contrast, other studies have shown no inflammatory response related to pathological tendons.^43^

The levels of MMP‐1 increased with loading, correlating with previous studies, which have shown increased MMP‐1 expression in tendinopathic^44^, ^45^ or ruptured^46^ tendons. Further, MMP‐1 expression has recently been shown to increase with overload fatigue inducing exercise in equine tendons.^47^ Interestingly, the increased levels of MMP‐1 in the IFM colocalized with staining for the C1,2C marker that detects the fragmented type I and II collagen, indicating that the MMP‐1 present is in an active form.

The up‐regulation of MMP‐3 and MMP‐13 in the current study also agrees with previous work, showing up‐regulation of both these MMPs in pathological tendons.^45^, ^47^, ^48^, ^49^, ^50^ However, MMP‐3 has also been shown to be down‐regulated in both ruptured and tendinopathic tendons, as shown by the mRNA expression level.^46^ The difference in response may be attributed to both timing (post‐injury) and the method used for identification of tissue response.

Interestingly, in this study, both inflammation and damage‐induced matrix remodeling seem to be concentrated in or in the vicinity of the IFM. This could be partially explained by the high number of cells present in the IFM as compared to fascicles, with more cells available to respond to overload of the IFM. However, it is also possible that IFM cells and tenocytes differ in their phenotype and activity levels. Their respective roles in initiation, progression, and resolution of inflammation, and the consequences for tendinopathy are yet to be elucidated.

Further, the mechanical role of IFM in tendon extension remains unclear. However, it has been hypothesized to be instrumental in allowing fascicles to slide with respect to each other,^11^ allowing higher tendon deformations before the onset of fascicle damage. In physiological use, the IFM may thus be subjected to higher shear stresses then the fascicular matrix, and as a sparse, softer component of tendon, may be more prone to damage and injury. This could partially explain the higher turnover rates in this phase as compared to fascicular matrix (observed high levels of MMP‐1 and C1,2C in control samples). IFM may need to turnover more rapidly and respond more robustly to damage to ensure proper IFM and tendon function. Further characterization of the mechanobiology of the IFM is needed to fully understand its role.

## Authors' Contribution

All authors have substantially contributed to research design, or the acquisition, analysis or interpretation of data, drafting the paper or revising it critically and have approved of the submitted version.
